# Congestive heart failure as a rare presentation of intratumoral bleeding of giant hepatic hemangioma: a case report and literature review

**DOI:** 10.1097/RC9.0000000000001015

**Published:** 2026-10-07

**Authors:** Zeki A. Abubeker, Zeru A. Sambi, Yonas T. Habtemariam

**Affiliations:** a Department of Surgery, Hepatopancreatobiliary Surgery Unit, Tikur Anbessa Specialized Hospital, Addis Ababa University, Addis Ababa, Ethiopia; b Department of Surgery, Hepatopancreatobiliary Surgery Unit, Hayat Hospital Medical College, Addis Ababa, Ethiopia; c Department of Surgery, Bahir Dar University College of Medicine and Health science, Bahir Dar, Ethiopia

**Keywords:** case report, cavernous hepatic hemangioma, congestive heart failure, giant hepatic hemangioma, intratumoral bleeding, severe anemia

## Abstract

**Introduction:**

Cavernous hepatic hemangiomas are the most common solid benign liver tumors and are usually asymptomatic. Rarely, they can present with fatal complications such as rupture and bleeding. This case highlights that a common benign tumor can present with life-threatening complications and that prompt diagnosis and treatment can be lifesaving.

**Case presentation:**

A 45-year-old man presented with right-upper-quadrant abdominal pain, progressive dyspnea, and bilateral lower-limb swelling of 2 weeks’ duration. Clinical examination revealed signs of congestive heart failure and severe anemia. Contrast-enhanced computed tomography (CECT) demonstrated a giant hepatic hemangioma with intratumoral hemorrhage involving the right lobe of the liver. After stabilization with multiple blood transfusions, the patient underwent surgical resection. Histopathology confirmed a cavernous hepatic hemangioma with hemorrhagic changes. The postoperative course was uneventful.

**Clinical discussion:**

Intratumoral hemorrhage in hepatic hemangiomas is exceptionally rare but can have serious sequelae. In this case, the bleeding resulted in severe anemia, likely precipitating congestive heart failure – an outcome not previously reported. Imaging studies are essential for diagnosis, while invasive diagnostic procedures should be avoided whenever possible. Surgical resection is the definitive treatment for hemangiomas that are complicated by hemorrhage.

**Conclusions:**

Giant hepatic hemangiomas can rarely present with intratumoral bleeding, leading to severe anemia and congestive heart failure. Early diagnosis and appropriate surgical intervention are crucial in preventing a *grave* outcome.

## Introduction

Cavernous hepatic hemangiomas are the most common benign solid liver tumors, accounting for up to 70% of benign hepatic lesions, with a prevalence of 3–20% and a female predominance^[^1,2^]^. Diagnosis is primarily made using ultrasonography (US), computed tomography (CT), or magnetic resonance imaging (MRI). Most hemangiomas are small, asymptomatic, and require no treatment. However, giant hemangiomas (>10 cm) may cause pain and rare but serious complications, including intratumoral hemorrhage, rupture, consumptive coagulopathy, and severe anemia, necessitating intervention^[^1^]^.

HIGHLIGHTS

- A giant hepatic hemangioma can rarely present with intratumoral hemorrhage, leading to life-threatening anemia.
- Severe anemia may precipitate congestive heart failure as an initial manifestation of a complicated hemangioma.
- Radiologic findings of intratumoral bleeding within a hemangioma can mimic malignant hepatic lesions, posing diagnostic challenges.
- Multidisciplinary management is crucial for stabilization, accurate diagnosis, and timely definitive treatment.
- Surgical resection remains a definitive and reliable treatment option for patients with complicated giant hemangiomas.

Herein, we report a rare and challenging case of a giant hepatic hemangioma complicated by intratumoral bleeding, resulting in severe anemia and congestive heart failure (CHF).

This case is reported in accordance with the SCARE 2025 guidelines^[^3^]^.

## Case presentation

A 45-year-old man presented with a 2-week history of right upper quadrant abdominal pain, dyspnea, fatigue, and bilateral lower-limb swelling. He had no history of trauma, gastrointestinal bleeding, hypertension, cardiac disease, smoking, alcohol use, or regular medication use.

On examination, he was in respiratory distress with a blood pressure of 110/60 mmHg, a pulse rate of 130 beats/min, a respiratory rate of 30 breaths/min, and a BMI of 27 kg/m^2^. He had marked conjunctival pallor, an S3 gallop, massive hepatomegaly extending to the umbilicus, and bilateral pitting pedal edema.

Laboratory investigations revealed severe microcytic anemia (hemoglobin, 5.8 g/dL; MCV, 53 fL) with normal leukocyte and platelet counts. Liver function tests were within normal limits. Renal function was normal, with a serum creatinine level of 0.6 mg/dL and a blood urea nitrogen level of 12 mg/dL.

Abdominal ultrasound demonstrated a large, well-defined, heterogeneous intrahepatic lesion with mixed echogenicity and cystic areas. A CECT of the abdomen demonstrated a large, well-defined hepatic mass measuring approximately 19 × 17 × 13 cm, involving the right posterior section and showing discontinuous peripheral nodular enhancement in the arterial phase with partial centripetal fill-in. However, most of the lesion remained hypodense during the portal venous and delayed phases. The absence of imaging features suggestive of malignancy, such as arterial hyperenhancement with washout, vascular invasion, infiltrative growth, mural nodularity, or metastatic disease, together with the presence of peripheral nodular enhancement and incomplete centripetal fill-in, favored the diagnosis of a giant hepatic hemangioma complicated by intralesional hemorrhage (Fig. 1).

**Figure 1.**
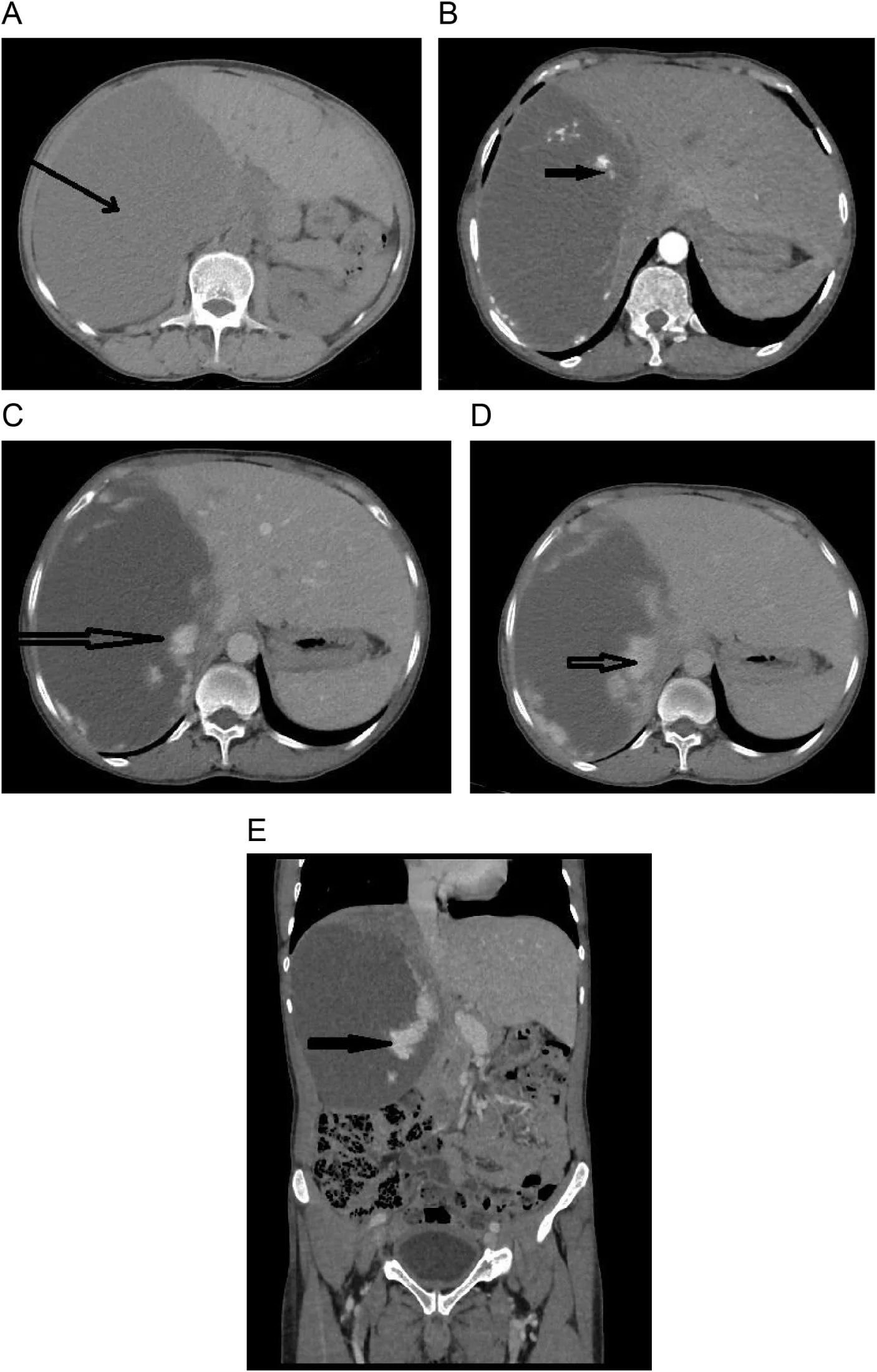
Abdominal CT. (A) Precontrast phase, axial view showing a huge hemangioma (black arrow) in the right hepatic lobe (hypodense relative to liver tissue). (B) Arterial phase, axial view showing peripheral nodular enhancement (black arrow). (C) Portal venous phase, axial view showing progressive central filling of the lesion (black arrow). (D) Delayed phase, axial view showing progressive central filling of the lesion (black arrow). (E) Coronal view showing the lesion in the right posterior section (black arrow).

Transthoracic echocardiography demonstrated left ventricular hypertrophy with a preserved left ventricular ejection fraction of 58% and no significant valvular abnormalities.

### Management and clinical course

Following multiple packed red blood cell transfusions, his hemoglobin increased to 9.7 g/dL, and 48 hours after admission, he underwent right posterior sectionectomy under general anesthesia with endotracheal intubation, through a Makuuchi incision. Intraoperatively, a giant hemorrhagic hemangioma with cystic degeneration was identified and resected after ligation of its feeding vessels (Fig. 2). Four Pringle maneuvers were applied during parenchymal transection. The operative time was 4 hours, with an estimated blood loss of 700 mL. Histopathology confirmed cavernous hemangioma with intratumoral hemorrhage (Fig. 3). Postoperative analgesia consisted of intravenous paracetamol and pethidine. Unfractionated heparin was initiated 24 hours postoperatively. The postoperative course was uneventful. The patient was discharged on postoperative day 5 and remained asymptomatic, with complete resolution of heart failure symptoms at the 7-month follow-up (Table 1).

**Figure 2.**
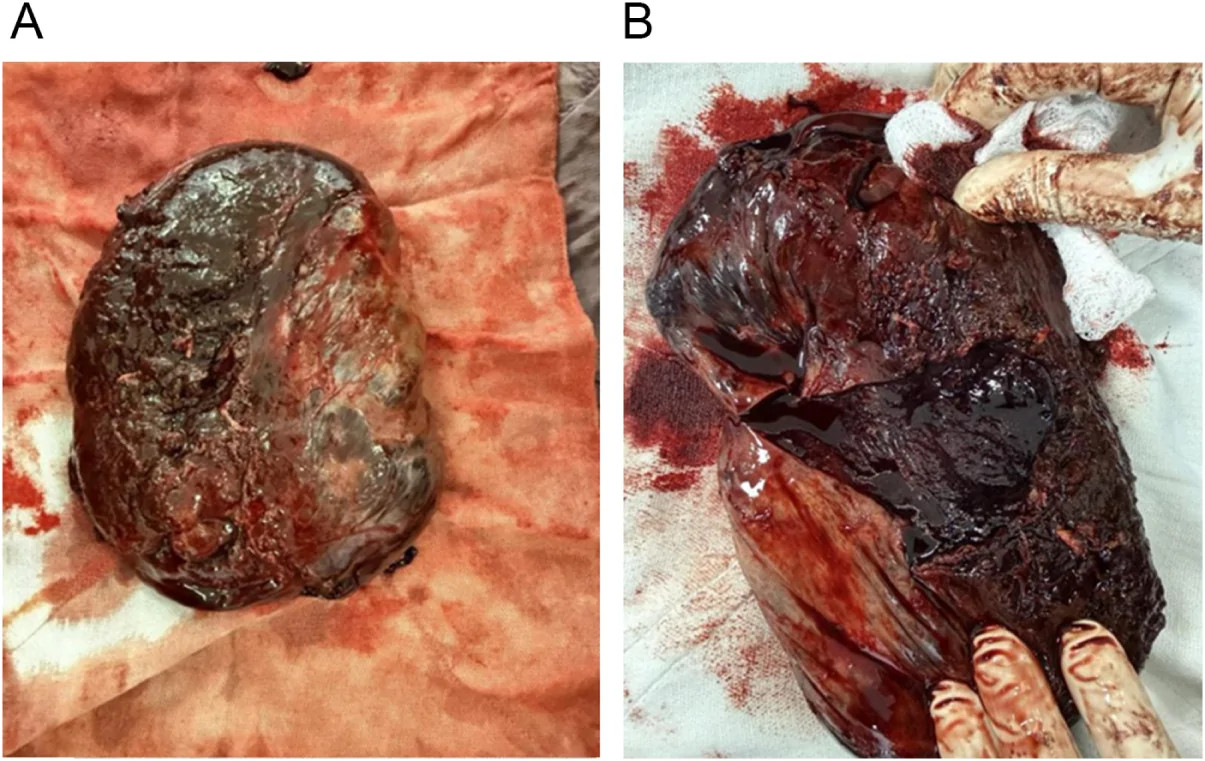
Intraoperative images. (A) Intraoperative photograph demonstrating the excised hepatic hemangioma specimen. (B) Intraoperative image showing a hematoma within the hepatic hemangioma.

**Figure 3.**
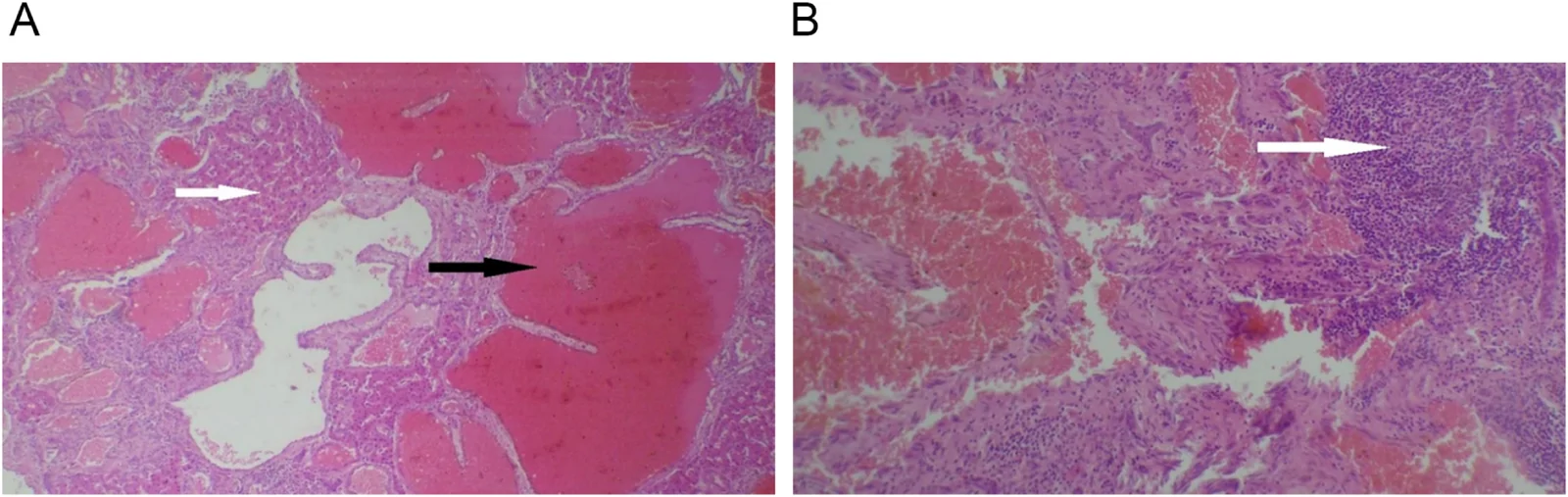
Histopathology. (A) Microscopy: Large-sized blood vessels (black arrow) interspersed with hepatocytes (white arrow). (B) Microscopy: Large-sized blood vessels and lymphoid cells (white arrow).

**Table 1:** Chronological sequence of the patient’s clinical progress.

Time point	Clinical event
Day 0	Onset of symptoms (e.g., abdominal pain, fatigue, leg swelling etc.)
Day 15	Presentation to hospital and initial clinical evaluation
Day 15	Laboratory investigations revealed severe anemia
Day 15	Blood transfusions started
Day 16	Contrast-enhanced CT scan performed demonstrating giant hepatic hemangioma
	Echocardiography performed showing
Days 15 and 16	Blood transfusions administered and patient optimized for surgery
Day 17	Surgical intervention performed (right posterior sectionectomy)
Postoperative days 1–5	Postoperative recovery and monitoring
Postoperative days 5	Patient discharged in stable condition
1 month	Outpatient follow-up with clinical improvement and/or normalization of hemoglobin
7 months	Outpatient follow-up with the patient remaining clinically stable

## Discussion and literature review

### Epidemiology and pathogenesis

Cavernous hepatic hemangiomas arise from mesenchymal tissue. The etiology remains incompletely understood; however, abnormal vasculogenesis and dysregulated angiogenesis are believed to play a central role in their development^**[**^4^**]**^. They are typically solitary, small, asymptomatic, and incidentally detected on imaging^[^1^]^. A marked female predominance (4:1–6:1) suggests a role for estrogen in their development^[^2^]^. Uncommonly, metoclopramide use has been associated with diffuse hemangiomatosis^[^5,6^]^. Hemangiomas measuring >10 cm are classified as giant hemangiomas^[^2^]^.

### Complications of giant hepatic hemangiomas

Most hemangiomas remain asymptomatic. Complications are related to tumor size, location, and internal changes such as hemorrhage or thrombosis^[^1^]^. Large hemangiomas located in the left hepatic lobe may exert a mass effect on the stomach, leading to symptoms such as dyspepsia, nausea, or early satiety. Lesions of substantial size may exert pressure on the biliary tree, hepatic veins, portal vein, or inferior vena cava, resulting in obstructive jaundice, portal hypertension, or edema in the lower limbs, respectively^[^2^]^.

### Rupture and hemorrhage

Hemorrhage is the most serious complication and may occur spontaneously or after trauma, anticoagulation, hepatocellular carcinoma, liver adenoma, or Kasabach–Merritt syndrome^[^4^]^. Although spontaneous rupture is rare (1–4%), it carries a mortality rate of 36–39%^[^2,7–9^]^. Acute hemorrhage presents with abdominal pain, hypotension, and hemorrhagic shock, whereas chronic intratumoral bleeding may cause progressive anemia, low-grade fever, or an enlarging abdominal mass^[^7,10^]^.

Severe anemia may lead to CHF by significantly reducing the blood’s oxygen-carrying capacity. To maintain adequate systemic oxygen delivery, the heart compensates through increased cardiac output, characterized by elevated heart rate and stroke volume, resulting in a hyperdynamic circulatory state. Persistent hemodynamic stress leads to ventricular hypertrophy and chamber dilation, ultimately progressing to myocardial dysfunction and heart failure^[^11^]^.

### Uniqueness of the present case

Intratumoral hemorrhage of hepatic hemangioma is exceptionally rare, with only nine cases reported in the literature^[^7^]^. Our patient presented with clinical features suggestive of congestive heart failure secondary to severe anemia resulting from intratumoral bleeding within a giant hepatic hemangioma. To our knowledge, based on our review of the available literature, no previous reports have described congestive heart failure as the presenting manifestation of intratumoral hemorrhage in a hepatic hemangioma, thereby expanding its recognized clinical spectrum.

### Diagnostic considerations

Hepatic hemangiomas can be diagnosed with high accuracy (>90%) using imaging modalities including US, CECT, MRI, and angiography^[^12^]^. On US, hemangiomas classically appear as homogeneous, hyperechoic lesions with well-defined margins and posterior acoustic enhancement.

On CT imaging, typical features include hypoattenuation on non-contrast scans, peripheral nodular enhancement during the arterial phase, progressive centripetal filling, and persistent enhancement on delayed phases, whereas intratumoral hemorrhage appears as high-attenuation areas on non-contrast CT. Giant hemangiomas may demonstrate atypical imaging findings, such as heterogeneous enhancement with marked central areas corresponding to thrombosis, extensive hyalinization, and fibrosis^[^2^]^.

The differential diagnosis includes intracapsular hemorrhage of biliary cystadenoma, hepatic cysts, and hepatic malignancies. MRI is particularly valuable in such equivocal cases. Hemangiomas typically appear hypointense on T1-weighted images and markedly hyperintense on heavily T2-weighted sequences. Intratumoral hemorrhage is characterized by high signal intensity on both sequences^[^12^]^.

Since imaging techniques are effective in diagnosing liver hemangiomas in the majority of cases, it is advisable to avoid liver biopsies whenever possible due to the associated risk of significant bleeding^[^10^]^.

### Management strategies

Given the high prevalence of hepatic hemangiomas and the extremely low incidence of complications, prophylactic surgery is not recommended. Asymptomatic patients, regardless of tumor size, require no treatment, and there is no evidence to support lifestyle restrictions, avoidance of pregnancy, or discontinuation of oral contraceptives solely due to the presence of a hemangioma^[^2^]^.

Treatment is indicated for severe or progressive symptoms, complications (e.g., hemorrhage, Kasabach–Merritt syndrome, or organ compression), inability to exclude malignancy, or significant patient anxiety^[^1,2^]^. Other causes of abdominal pain should be excluded before attributing symptoms to a hemangioma.

### Therapeutic options and rationale for surgery

Management options include surgical resection, transarterial embolization (TAE)/transarterial chemoembolization (TACE), ablative therapies, and, rarely, liver transplantation or pharmacologic therapy such as sorafenib^[^12,13^]^. Although TAE and TACE can be considered in selected stable patients, surgical resection appears to be the preferred treatment for complicated hemangiomas^[^4,14^]^. Surgical resection remains the definitive treatment for complicated hemangiomas. Enucleation is an ideal surgical management option for peripherally located lesions, whereas tumors nested deep within the hepatic parenchyma are more safely treated with anatomic hepatic resection^[^14^]^. Liver transplantation is reserved for exceptional cases, such as diffuse hemangiomatosis and technically unresectable^[^2^]^.

Our case represents the tenth reported instance of an intratumoral hemorrhage in a hepatic hemangioma and was successfully managed with surgical resection.

## Conclusion

Although cavernous hepatic hemangiomas are common and benign, they can present with life-threatening complications such as intratumoral hemorrhage, which can result in severe anemia and congestive heart failure. This case highlights the importance of clinicians recognizing such atypical presentations and supports surgical resection as the most reliable definitive treatment for hemangiomas complicated by intratumoral bleeding.

## Data Availability

Not applicable.
